# An Adaptive Application-Aware Dynamic Load Balancing Framework for Open-Source SD-WAN

**DOI:** 10.3390/s25175516

**Published:** 2025-09-04

**Authors:** Teodor Petrović, Aleksa Vidaković, Ilija Doknić, Mladen Veinović, Živko Bojović

**Affiliations:** 1 Faculty of Informatics and Computing, Singidunum University, 11000 Belgrade, Serbia; tpetrovic@singidunum.ac.rs (T.P.); mveinovic@singidunum.ac.rs (M.V.); zbojovic@singidunum.ac.rs (Ž.B.); 2 Faculty of Sciences, University of Novi Sad, 21000 Novi Sad, Serbia; doknicilija98@gmail.com

**Keywords:** SD-WAN, dynamic load balancing, application-aware networking, open-source SDN, performance optimization

## Abstract

Traditional Software-Defined Wide Area Network (SD-WAN) solutions lack adaptive load-balancing mechanisms, leading to inefficient traffic distribution, increased latency, and performance degradation. This paper presents an Application-Aware Dynamic Load Balancing (AADLB) framework designed for open-source SD-WAN environments. The proposed solution enables dynamic traffic routing based on real-time network performance indicators, including CPU utilization, memory usage, connection delay, and packet loss, while considering application-specific requirements. Unlike conventional load-balancing methods, such as Weighted Round Robin (WRR), Weighted Fair Queuing (WFQ), Priority Queuing (PQ), and Deficit Round Robin (DRR), AADLB continuously updates traffic weights based on application requirements and network conditions, ensuring optimal resource allocation and improved Quality of Service (QoS). The AADLB framework leverages a heuristic-based dynamic weight assignment algorithm to redistribute traffic in a multi-cloud environment, mitigating congestion and enhancing system responsiveness. Experimental results demonstrate that compared to these traditional algorithms, the proposed AADLB framework improved CPU utilization by an average of 8.40%, enhanced CPU stability by 76.66%, increased RAM utilization stability by 6.97%, slightly reduced average latency by 2.58%, and significantly enhanced latency consistency by 16.74%. These improvements enhance SD-WAN scalability, optimize bandwidth usage, and reduce operational costs. Our findings highlight the potential of application-aware dynamic load balancing in SD-WAN, offering a cost-effective and scalable alternative to proprietary solutions.

## 1. Introduction

Today’s enterprise networks increasingly rely on high-performance and resilient network architectures to support the demands of cloud applications, remote workforces, and globally distributed operations. Traditional Wide Area Networks (WANs), which typically utilize technologies such as Multi-Protocol Label Switching (MPLS) or leased lines, meet significant challenges in adapting to the dynamic nature of modern application traffic. These legacy solutions often lack agility, are expensive to scale, and exhibit inefficiencies in traffic management across heterogeneous links. To overcome these issues, organizations are adopting SD-WAN [1], which enables dynamic routing and intelligent traffic management across multiple WAN links. This way, it is possible to build a more flexible and cost-effective solution using the principles of SDN networking. SD-WAN provides centralized management of traffic across different WAN links (e.g., MPLS, broadband, 4G, 5G, and 6G) and enables dynamic traffic routing based on real-time conditions. This approach improves application performance, reduces costs, and improves user experience [2]. 

Existing (proprietary) SD-WAN solutions offer robust performance and comprehensive feature sets but are typically closed-source, limiting customization and adaptability. However, they have several limitations that affect their effectiveness. Static load balancing is a problem because most SD-WAN implementations use predefined traffic policies (static load balancing) that cannot adapt dynamically [3]. This means that even when changes occur in the network, the system cannot dynamically redistribute traffic based on current conditions, which can lead to congestion and suboptimal resource utilization. In addition, the closed nature of these solutions is a serious limitation, as many vendors require the use of their specific equipment and software (proprietary constraints), which limits user flexibility. Also, the lack of application awareness in existing load-balancing methods means that the system does not distinguish between different types of traffic [4]. This can lead to situations where critical applications (real-time applications) are not prioritized over less important types of traffic, resulting in reduced QoS.

In contrast, open-source SD-WAN solutions offer a flexible, customizable option to meet users’ needs [5]. Despite the many benefits that open-source solutions bring, they often lack advanced features like dynamic load balancing that can adapt to specific applications. This gap presents an opportunity to create smart systems that can adjust to changing network and application conditions. This paper presents such a solution in the form of an Application-Aware Dynamic Load Balancing (AADLB) algorithm designed to enhance traffic distribution in open-source environments. The algorithm dynamically optimizes routing decisions using real-time performance metrics (e.g., CPU and memory utilization, latency, and packet loss). It improves resource utilization and service quality while increasing infrastructure flexibility. From the scientific and methodological point of view, its contributions are as follows:

- An adaptive load-balancing framework that accommodates traffic routing based on real-time network and application metrics.
- Implementation in an open-source SD-WAN environment, ensuring cost-effective and scalable deployment.
- Comprehensive performance evaluation demonstrates a 7.09% latency reduction and a 9.80% improvement in RAM efficiency compared to traditional approaches.

Our solution improves resource utilization and service responsiveness by incorporating real-time metrics and application-aware intelligence. Commercial SD-WAN solutions have limitations, highlighting the need for adaptive mechanisms for real-time network conditions. We propose a heuristic-based load-balancing algorithm that dynamically adjusts routing based on performance metrics, offering an effective open-source alternative.

## 2. Related Work

Modern enterprise networks require continuous traffic monitoring and intelligent mechanisms to adapt traffic flows dynamically across heterogeneous infrastructures. This is vital for optimal network performance and congestion prevention [6]. SD-WAN technologies have emerged as a promising approach to optimizing connectivity between remote offices, cloud services, and data centers. While proprietary SD-WAN solutions provide robust performance and a rich feature set, they often lack adaptability and granular visibility into application-specific and host-level metrics. Most of them are expensive and inflexible, especially for small and medium-sized enterprises. Open-source SD-WAN solutions offer an affordable option that reduces the risk of vendor lock-in. The analysis of their designs looks at three main areas: traffic engineering, load balancing, and adaptive routing.

### 2.1. Proprietary vs. Open-Source SD-WAN Platforms

Proprietary SD-WAN platforms provide several advantages that make them suitable for large organizations with demanding network environments. Primarily, they offer advanced network traffic optimization functionalities, such as dynamic load balancing, QoS, and intelligent traffic routing, which enables better resource distribution and improves application performance [7]. These platforms can integrate with many security features, including advanced threat protection (IPS), VPN encryption, content filtering, and antivirus protection, thus providing a high level of data protection. Centralized management through platforms such as FortiManager for Fortinet [8] or vManage for Cisco [9] simplifies network setup, configuration, and monitoring.

This enables administrators to manage network policies and devices from a single location. Scalability is also a significant benefit, as proprietary SD-WAN solutions like Cisco SD-WAN and VeloCloud (VMware SD-WAN) can easily grow with the organization [10]. They support the integration of new locations—including multi-cloud environments—as well as additional devices and functionalities, all without significant complexity.

Proprietary SD-WAN platforms also have certain constraints related to high costs (e.g., licensing, hardware, and maintenance), implementation complexity (can require highly specialized knowledge and technical expertise), and limited flexibility (can increase the time needed for setup and potentially introduce errors) [11]. In many cases, there is also the risk of a “lock-in” effect, where organizations become tied to a single vendor, which can make it difficult to change solutions in the future.

Here, we want to point out the constraints of proprietary platforms, which is especially important in the context of our research. Proprietary SD-WAN platforms offer built-in load-balancing features, but they often use fixed rules. These rules do not change in response to real-time network performance or specific application needs. Cisco SD-WAN features a centralized control plane, vSmart, which enables application-aware routing through its Application-Aware Routing (AAR) and Control Policy Framework. These systems allow for choosing paths based on Service-Level Agreement (SLA) compliance. However, they usually rely on fixed thresholds and do not adjust in real time to metrics like CPU and memory usage at the host level. Using the vManage platform for configuration requires much manual work. This can lead to a slower response when faced with unexpected surges in traffic or delays within the system [9].

In VMware SD-WAN, the system utilizes Dynamic Multi-Path Optimization (DMPO) to evaluate link performance based on metrics such as jitter, latency, and packet loss. However, DMPO does not consider the current load in application instances or the degree to which the servers are being utilized. This can lead to variable traffic distribution when many users are online or when backend services have different processing abilities. This issue is particularly relevant for applications that require low latency, such as VoIP or real-time video [10]. Also, there are limitations such as a lack of visibility into server utilization and responsiveness and susceptibility to path flapping caused by minor metric fluctuations.

Fortinet SD-WAN is managed via FortiManager and supports WAN path steering based on SLA and link-health thresholds. The path selection process mainly focuses on link-state parameters and does not incorporate real-time performance data from backend nodes. This can cause resource bottlenecks. This is evident, especially when traffic is sent to overloaded instances during uneven load situations or recovery from failures. Without visibility into host-level metrics, Fortinet SD-WAN may struggle to redistribute traffic effectively among application endpoints.

These constraints clearly indicate the absence of fine-grained, application-aware traffic distribution mechanisms that can dynamically adapt to heterogeneous runtime conditions. The lack of dynamic weight assignment based on actual resource availability and real-time connection statistics results in suboptimal system responsiveness. This implies increased latency and potential performance degradation, particularly in distributed, cloud-centric environments. For this reason, we decided to focus our research on building a heuristic-driven routing framework that will introduce dynamic, metric-driven load balancing in SD-WAN. Our main idea was to incorporate host-level metrics (CPU, memory usage, and resource trends) and connection-level metrics (average connection duration and data volume) into the routing logic and to ensure more efficient traffic steering, minimize congestion, and maintain consistent quality of service even in unstable network conditions.

Unlike proprietary platforms, open-source SD-WAN platforms represent a cost-effective and highly customizable alternative. They represent a good solution for organizations with limited budgets to avoid the expensive licensing of proprietary solutions [12]. The additional benefit is the opportunity to configure them according to specific needs. Moreover, these platforms make it possible to integrate with different network infrastructures and add new functionality; with strong support from the open-source community, updates and security patches are often available through collaborative development, and users can contribute to software improvements and solve specific problems through code modifications.

Open-source SD-WAN platforms (e.g., OpenWrt, VyOS, and pfSense) provide significant flexibility and cost savings. However, they often lack the advanced features required for fine-grained traffic management and dynamic load balancing. OpenWrt primarily focuses on routing and firewall configuration and provides basic load balancing through static rules or round robin methods. VyOS supports policy-based routing and firewalling but requires extensive manual configuration for load balancing and lacks dynamic path optimization features. pfSense, although robust in terms of firewalling and VPN support, provides limited scalability and lacks deep integration with real-time telemetry or application-aware metrics. Generally, open-source SD-WAN solutions have constraints related to complex implementation, lack of support, and potential instability and scalability issues. Most of them use static or session-based load balancing, which does not respond to runtime performance metrics. There is no support for host-level monitoring within routing logic, and manual intervention or scripting to adjust policies is required, increasing complexity and potential for misconfiguration. Finally, scalability can be limited, as some open-source solutions are not optimized for large networks with hundreds of nodes and require additional configuration to achieve the same performance as proprietary alternatives. Bearing in mind these facts, we focused our research on building an open-source framework, which would include real-time telemetry, heuristic weight generation, and dynamic traffic steering to offer enterprise-grade functionality on top of open-source platforms. Our goal was to embed connection-level awareness and resource utilization feedback into the routing logic. In Table 1, we provide a summary of the advantages and constraints of existing SD-WAN platforms.

### 2.2. Load Balancing in SD-WAN

Enterprises are increasingly adopting innovative business strategies. To realize these strategies and stay competitive, they must implement emerging technologies (e.g., cloud computing, Software As a Service (SaaS), mobility, network security, and unified communications). Cloud technology plays an important role because it offers seamless data transfer across various locations, efficient storage solutions, and other advantages. However, cloud adoption introduces new challenges in traffic distribution across branches, data centers, and cloud resources, potentially leading to bottlenecks and inefficient network utilization. The authors in [13] describe in detail the implementation of various load-balancing techniques in SD-WANs, including performance optimization methods, such as those proposed in [14]. They use session-based and per-packet load-balancing methods and connect theoretical concepts with real-world challenges in implementing SD-WAN solutions. This makes their work useful for designing and optimizing network performance. Further, they explain how SD-WAN enables better utilization of network resources and reduces latency issues, which is crucial for improving network performance and reliability. The text highlights the scalability and flexibility of SD-WAN technology in WAN networks. Although it mentions performance optimization through load balancing, it lacks a thorough analysis of specific cases and quantitative results, which would better evaluate its effectiveness. The research offers useful technical guidance, but the lack of concrete case studies or implementations in real networks in specific applications may limit its applicability in practice.

SD-WAN represents a modern network solution that uses SDN principles to manage WANs, with the controller that acts as a network gateway [15]. VyOS is one of the open-source platforms that can be used as a base for SD-WAN implementations, offering flexibility and extensibility [16]. Therefore, centralized control helps enterprises optimize traffic across WAN links like MPLS, broadband, LTE, and 5G. In [17], the authors propose an improved SD-WAN architecture, which introduces a traffic monitor between the control plane of the edge routers and the central SD-WAN controller. This monitor uses Deep Packet Inspection (DPI) to analyze network traffic dynamically and detect changes in the performance of traffic flows. When the monitor detects network traffic congestion or degradation, it sends information to the SD-WAN controller, which then dynamically reroutes flows and optimizes network load. The proposed solution combines classic load-balancing algorithms (round robin, least connections, source IP hash, least bandwidth, and equal-cost multi-path routing) with real-time monitoring to improve the performance and stability of SD-WAN networks. Although it enhances traffic distribution and reduces latency, this solution requires a large amount of CPU and RAM resources, which can lead to overloading with many simultaneous flows. Also, the system generates a large amount of traffic data, which can overload the controller if used in large network environments. Comparative evaluations of various SD-WAN platforms, such as those presented in [18], also highlight the trade-offs between performance, scalability, and security features.

Some authors propose in their research the application of AI and machine-learning solutions for optimizing load balancing in SD-WAN networks. In [19], the authors use the Deep Reinforcement Learning (DRL) approach for this purpose. Specifically, they use the Multi-Agent Deep Q-Network (MADQN) strategy to redistribute network traffic among SD-WAN controllers efficiently. They solve the problem of SD-WAN controller overload, which occurs due to limited processing capacity and many services passing through the network. The proposed model uses neural networks to analyze the current state of the network and dynamically select the optimal path for traffic redirection, with the aim of minimizing latency and the cost of traffic migration between controllers. The proposed Deep Reinforcement Learning (DRL) model optimizes traffic distribution. This way, it is possible to reduce congestion and improve network performance by adjusting decisions to lower data transfer costs, allowing controllers to respond effectively to sudden traffic changes. However, the proposed DRL method requires a lot of processing power (CPU) and memory (RAM) for data processing and model training and is complex to implement. Furthermore, DRL models often require a long training period before they achieve optimal performance.

One key reason for using SD-WAN in modern network infrastructure solutions is the multi-cloud approach. SD-WAN enables better traffic management and connectivity between multiple cloud platforms (e.g., AWS, Microsoft Azure, or Google Cloud) and local networks. In [20], the authors propose a solution for better optimization of network traffic, which is based on Dynamic Traffic Management (DTM) in SD-WAN networks. The main goal is to optimize traffic transfer between different clouds and reduce the cost of using inter-domain links. The proposed solution facilitates dynamic traffic redirection from congested links to those with lower congestion, thereby optimizing resource utilization and minimizing traffic transmission costs. Instead of centralized control, it implements a distributed logic that allows collaboration between different service providers without the need for a central control system. There are several drawbacks, notably its reliance on cooperation among various ISPs. This collaboration can be difficult to achieve due to differing business interests and competition.

Additionally, the implementation and integration within heterogeneous networks can be quite complex. Scalability can become problematic in larger networks, and increasing the number of connected cloud services can lead to security and performance monitoring challenges. Also, investment in traffic optimization infrastructure is required, which can increase costs, while a distributed approach can make it difficult to monitor and manage network resources dynamically.

We focus our research on optimizing application-specific traffic management, leveraging heuristics for dynamic routing decision making. Incorporating real-time metrics and specific application requirements will enhance the assessment of the proposed solutions’ effectiveness.

## 3. System Design and Methodology

**Figure 1:**
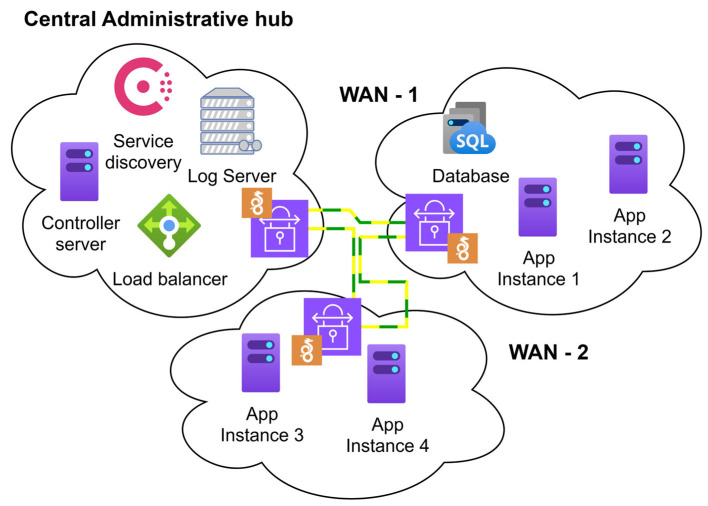
Design of the SD-WAN system.

The environment used in this research consists of three independent WAN networks, each located in a separate geographic location. These networks are interconnected using WireGuard VPN tunnels, creating a unified communication network that allows each network and its machines to maintain a direct connection to all other networks in the system. This setup ensures seamless communication among all machines within the three networks, whether with unrestricted or restricted access (Figure 1).

The setup depicted in Figure 1 enables seamless communication within each network, with the Central Administrative Hub playing a pivotal role. This network houses critical servers responsible for managing and maintaining the overall system. These include the controller, which orchestrates system-level operations, a log server for collecting and processing system and network activity data, a service registry for maintaining real-time application instance records, and a load balancer for distributing traffic. These components form the backbone of the environment, ensuring proper functionality and data flow across the system.

The second and third networks, WAN-1 and WAN-2, are dedicated to hosting application services and resources. WAN-1 is primarily used to host backend APIs, databases, and other services that require low latency and high responsiveness. This network is closer to end users and critical services. WAN-2, on the other hand, is configured to provide redundancy and host less critical services, supporting failover scenarios and load distribution testing. Together, WAN-1 and WAN-2 create a distributed environment that mimics real-world resource placement and ensures the robustness of the load-balancing algorithm, a key factor in the system’s high performance and adaptability.

Each machine running a service application instance is equipped with dedicated monitoring tools that continuously collect real-time host and connection metrics. The metrics server continuously collects these data, which are subsequently processed by our dynamic weight-generating algorithm. Our custom framework’s ability to adapt to changing network situations not only enables efficient traffic routing but also highlights one of its key advantages: the ability to respond immediately to changes in load, thereby minimizing congestion and ensuring optimal system usage and performance.

This setup provides the foundational framework for testing the proposed load-balancing algorithm. WAN-1 and WAN-2 strategically distribute app instances, databases, and APIs, allowing the system to adapt traffic routing based on real-time metrics. This adaptability, a key feature of the system, ensures that it can perform effectively in varying conditions, confirming that the algorithm routes traffic effectively.

### 3.1. Adaptive Load-Balancing Scheme

**Figure 2:**
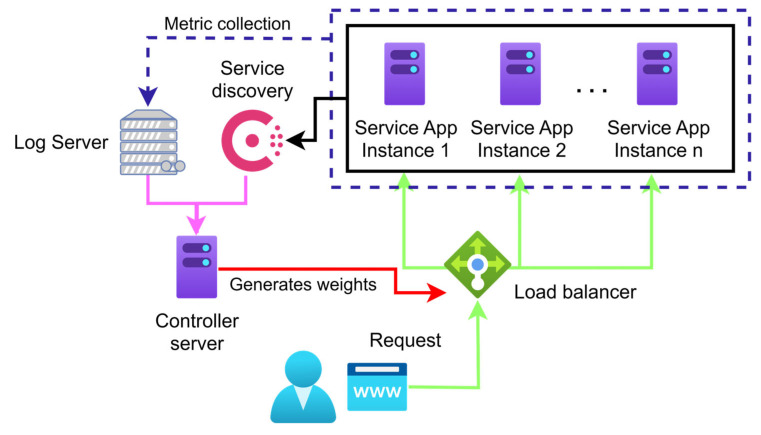
The scheme for dynamic load balancing.

The load-balancing scheme we propose (Figure 2) is designed to generate application-specific weights for each service registered in our service registry under a group service name. By using detailed host-level metrics (such as CPU, RAM, and their trends of change, whether the resource demand is increasing or decreasing) alongside connection-level metrics (including average connection duration, bytes sent/received, and error rates), our algorithm continuously adapts to real-time system conditions. This results in a more granular and responsive traffic routing that outperforms traditional static methods.

The load balancer then uses this registry to distribute traffic proportionally to each instance’s utilization rate. We derive these weights using previously mentioned real-time metrics. The system’s adaptability to network changes is crucial. It efficiently distributes load by assigning higher weight values to underutilized instances, allowing the Fabio load balancer to direct more traffic to them.

This adaptive approach optimizes traffic distribution by dynamically adjusting weight values based on real-time metrics. Algorithm 1 illustrates the detailed specifications of the proposed algorithm and offers a comprehensive breakdown of the weight generation process within our load-balancing framework.
**Algorithm 1**: Weight Generating Algorithm**  Input:** instances (application instances), metrics (CPU, memory, etc), weights (user-defined)**  Output:** weights (Fabio weights for each instance)  weights_config ← user-defined weight percentages
**  for** each instance i in instances **do**
    metrics ⟵get_all_metrics(i, last 1 min)
    Extract metrics:
      duration_avg ⟵get_connection_duration(metrics)      bytes_send ⟵avg_sent_bytes(metrics)      bytes_received⟵avg_received_bytes(metrics)      error_ratio ⟵connection_error_ratio(metrics)      trend ⟵calculate_trend(metrics)      normalized_metrics⟵normalize_all_metrics(…)      $weight\_score\;⟵\sum_{metric}\left(normalized\_metric\times weights\_config.metric\right)$      fabio_weight⟵map_to_Fabio(weighted_score)      update_Consul_weight(i, fabio_weight)
**  end for**

The initial weights for all application instances are arbitrarily set to equal values, ensuring fair traffic distribution at the start. Each 1 min interval triggers a complete recalculation of instance weights based exclusively on current real-time metrics without dependence on previously calculated weights. The recalculated weights guide the load balancer to direct new incoming requests preferentially toward instances with higher scores, indicative of lower resource utilization and greater availability. This proactive routing strategy neither places incoming requests on hold nor rejects them; instead, it dynamically redirects traffic to achieve optimal instance utilization, reduces latency, and maintains consistent service availability. The weight generation algorithm consists of the following steps:

- Data Acquisition and Metrics Derivation: The algorithm relies on two primary categories of data: Host-Level Metrics, which include CPU and RAM utilization and the trend coefficient, and Connection-Level Metrics, which include the average connection duration, average bytes sent and received, and connection error rate.
- Normalization: Each metric is normalized to a standard scale of 0–100. For instance, CPU utilization is inversely scaled. Slower utilization scores result in a higher normalized score. At the same time, connection metrics are normalized based on predefined ideal ranges and specific processing functions based on scenarios.
- Weighting and Scoring: Each metric is assigned a weight percentage, reflecting its importance in the final calculation. The normalized scores are multiplied by their respective weights to compute the weighted scores.
- Weight Mapping: The final weighted score is scaled to an integer and added as a tag to a specific app instance in our service registry. The higher the score, the more resources the instance has, and the more traffic it can handle. This enables the load balancer to allocate more traffic to application instances with more resources, i.e., less burdened ones, thereby optimizing our system’s performance.

### 3.2. Implementation

Our proposed dynamic load-balancing algorithm is implemented using a suite of open-source tools that collectively ensure optimal resource utilization, system performance, and traffic management in an SD-WAN. The HashiCorp Consul Service registry maintains a real-time registry of active application instances, while the lightweight Fabio load balancer uses this registry and the weights associated with each application instance to distribute traffic efficiently. Complementary tools, such as Zeek, Filebeat, Logstash, Elasticsearch, and Zabbix, are deployed to capture, process, and analyze both host-level and connection-level metrics dynamically to be used in the weight-generating portion of our algorithm.

All services run on dedicated Virtual Machines (VMs), ensuring a consistent testing environment and simplifying system maintenance. This setup guarantees that performance improvements are due to our algorithm’s efficiency, not hardware differences. This integration of real-time monitoring systems and adaptive traffic management is the core strength of our proposed model. It enables the system to dynamically balance load even under rapidly changing network conditions.

HashiCorp Consul, a robust service registry, maintains an up-to-date list of all active application instances under a shared service name. Fabio, a lightweight HTTP(S) router, retrieves the registry data from Consul and automatically applies the algorithm’s calculated weights for more competent traffic distribution. Zeek logger, a network traffic monitoring solution, captures network traffic information, such as connection durations, transferred bytes, and error rates. Filebeat service, a log-forwarding utility, transfers these logs to a centralized location. In contrast, Logstash, a versatile data processing service, applies custom filters and transformations to incoming logs. Elasticsearch, a distributed search and analytics engine, stores these refined metrics and makes them readily accessible for real-time queries by the algorithm. Meanwhile, Zabbix, a comprehensive monitoring solution, is used to gather host-level metrics like CPU and memory usage and trend coefficients for predicting resource availability. Combining these continuous data streams allows the dynamic load-balancing algorithm to update traffic weights instantly, ensuring the connections are always routed to the most capable application instance.

### 3.3. Performance Metrics

**Figure 3:**
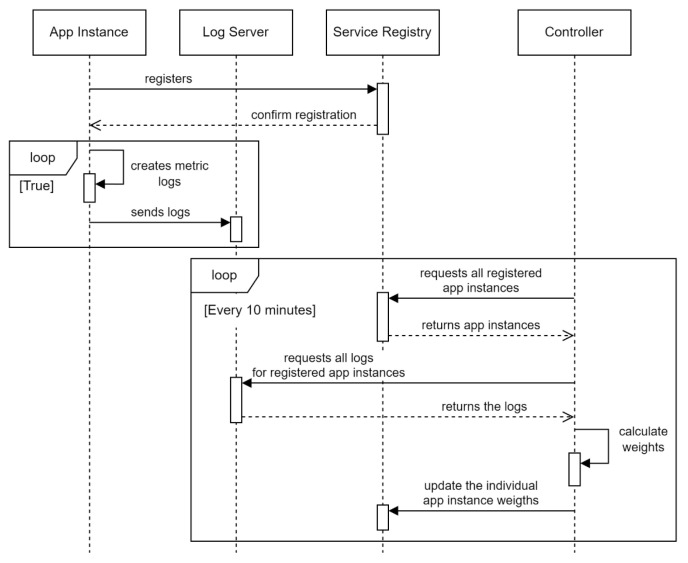
Dynamic load-balancing process: Sequence diagram.

Acquiring and deriving metrics for the dynamic weight-generating algorithm involves systematic, real-time collection, processing, and aggregation of host-level and connection-level data. These metrics evaluate the performance and utilization of application instances and are systematically processed to ensure a reliable and efficient load distribution system (Figure 3). 

The sequence diagram (data processing workflow) begins with systematically acquiring, processing, and aggregating metrics every 1 min. The first step is service instance discovery, a crucial process where all registered application instances for a specific service name are fetched from the Consul registry. This ensures that the algorithm operates with the latest available data regarding service instances. Then, for each of these services, we collect their associated metrics:

- Host-Level Metrics: CPU and memory utilization data are collected using a dedicated monitoring service (e.g., Zabbix). A key derived metric is the trend coefficient, calculated from current 1 min interval metrics grouped by service IP address or domain name, which indicates increasing or decreasing resource load to support proactive traffic redirection.
- Connection-Level Metrics: Real-time network traffic, including communication between the load balancer and application instances, is monitored via Zeek logs. Essential metrics are extracted from logs (e.g., conn.log, http.log), stored in Elasticsearch, and periodically queried to supply data for processing.

The key host- and connection-level variables referenced throughout the remainder of this section are summarized in Table 2.

After acquisition and initial processing, the collected metrics are combined to provide a holistic view of each application instance’s state. These consolidated data then serve as input for the subsequent stages: Score Normalization, Weight Assignment, and Weight Mapping. The stages are as follows:

- Score Normalization: This stage applies predefined functions to each metric, converting raw values into a standardized score. For host-level metrics like CPU and RAM utilization, scores are inversely proportional to their percentage (e.g., <10% utilization yields 100 points; 70% yields 30 points). Connection-level metrics such as average connection duration are mapped to ideal ranges (e.g., 0.1–0.2 s scoring 100 points), with scores proportionally reduced for values exceeding optimal thresholds based on custom formulae and user-defined cutoff values (e.g., a 0.3 s duration with a 0.2 s cutoff and 20-point penalty per 0.1 s interval results in 80 points). The connection error ratio is normalized to yield higher scores for lower error rates, incorporating specific user-defined cutoffs for ideal performance.
- Weight Assignment and Scoring: Metrics are weighted based on their importance to service needs, determined via a heuristic approach combining domain knowledge and expert assessment under real-environment conditions. Our own experience and detailed understanding of specific network traffic requirements further enhanced the decision-making process. For example,
  ○ CPU and RAM utilization are each assigned a 15% weight due to their direct impact on an instance’s traffic handling capacity.
  ○ The trend coefficient receives 20% for its role in anticipating resource demands.
  ○ Average connection duration, indicating service responsiveness, is assigned the highest weight at 20%.
  ○ Bytes sent and received are each weighted at 10%, providing network bandwidth insight.
  ○ The connection error ratio receives 10%, significant for identifying potential service failures.
- Weight Mapping: To ensure compatibility with Fabio’s weight system, the total weighted score for each application instance is rounded to the nearest integer (e.g., 56.5 points rounded to 57 points). This rounded value is then converted into a tag descriptor and attached to the corresponding application instance within its service group in Consul, enabling Fabio to dynamically distribute traffic.

Additionally, although data collected at the beginning of the interval might not directly influence immediate decisions due to their age, such historical data retain substantial value for future enhancements of the load-balancing algorithm. The Zeek logger generates approximately 20 different types of logs, providing extensive data resources that can be leveraged in future algorithmic improvements, enabling finer granularity and more responsive adaptation even on an application level.

## 4. Experimental Setup and Testing

### 4.1. Network Topology and Node Distribution

To evaluate the effectiveness of the proposed AADLB algorithm, we conducted experiments in a custom-built SD-WAN testbed designed to simulate realistic enterprise network conditions. The test environment included geographically distributed domains, diverse service placements, and mixed traffic types. This section details the experimental methodology, node distribution (described in Section 3), traffic models, and evaluation scenarios.

Different traffic types are generated to simulate enterprise workloads, including HTTP REST API calls targeting backend services, simulated WebRTC UDP packet streams for latency sensitivity analysis, and scheduled bulk data transfers to stress the database and bandwidth. This variety ensures that the network experiences realistic mixed traffic patterns and congestion conditions during evaluation.

This results in a network with seven main VMs for service deployment and three additional traffic generator nodes positioned externally. All nodes are connected through secure VPN tunnels and can exchange traffic without restrictions.

This setup represents a medium-density SD-WAN topology, with redundant application instances distributed across remote WANs and a central control domain. It is designed to reflect realistic enterprise-scale SD-WAN infrastructures where latency, load balancing, and service responsiveness are critical. This topological layout ensures that the testing environment provides sufficient complexity while remaining manageable and reproducible for evaluation purposes. The full node inventory is summarized in Table 3.

### 4.2. Virtual Infrastructure and Deployment Environment

The experimental environment was deployed on a high-performance physical server to ensure reliable evaluation of the proposed load-balancing algorithm under controlled and reproducible conditions. The infrastructure was hosted on a Supermicro SYS-7038A-I server equipped with dual Intel Xeon E5-2630 v4 CPUs, providing a total of 20 physical cores and 40 logical processors, along with 224 GB of DDR4 RAM. The system ran Windows Server 2025 Datacenter Edition, which offered robust virtualization capabilities and resource management.

We set up VMs using Hyper-V across three networks: Central Administrative Hub, WAN-1, and WAN-2. This setup helps ensure that resources mimic real business tasks, which prevents performance issues. The load balancer (Fabio) was hosted on a dedicated VM with four vCPU cores and 8 GB RAM, ensuring sufficient capacity to handle concurrent HTTP requests. The controller responsible for load-balancing logic and weight computation was deployed on a high-capacity VM with six vCPUs and 16 GB RAM. The central log server (running Filebeat, Logstash, and Elasticsearch) and service discovery server (Consul) were hosted on two additional VMs, each provisioned with four vCPUs and 8 GB RAM and two vCPUs and 4 GB RAM, respectively. WAN-1 hosted two backend service instances and a central MySQL database, each VM having two vCPUs and 2 GB RAM. WAN-2 hosted two more backend service instances with the same specifications for load distribution and failover. The deployment structure ensured the isolation of core service components, high availability, and realistic network segmentation for SD-WAN evaluation, reflecting real-world enterprise conditions with efficient, low-latency load distribution across sites.

### 4.3. Traffic Generation Model

To create a real enterprise network environment, we used a traffic generation model that included random timing and payload sizes. This model uses methods from earlier studies, such as the technique by Patil et al. [21], to simulate real-world service usage. Traffic was generated using a high-level synthetic traffic generator deployed on three external VMs. Each VM runs multiple independent threads that simulate user behavior and interaction patterns with backend services.

The traffic generation model incorporates the following patterns:

- Arrival Process: Each thread initiates a request batch after a randomized idle interval sampled from a uniform distribution over the interval [1, 20] s, effectively simulating asynchronous user activity and preventing traffic burst synchronization.
- Request Volume per Batch: The number of requests per batch is randomly chosen from a discrete uniform distribution in the range [10, 100], ensuring variability in burst sizes like real-world request patterns.
- Request Types: The generated traffic consists of HTTP REST API calls, including GET (≈70%), POST, INSERT, and UPDATE operations (≈30%). The traffic composition shows typical enterprise workloads, marked by mostly read-heavy operations, with write operations still placing significant demands on processing resources and bandwidth.
- Payload Characteristics:
  ▪ GET requests are usually simple and require small processing from the server.
  ▪ POST/INSERT/UPDATE requests include varied payload sizes, which are synthetically generated with sizes ranging from 256 bytes to 16 KB, based on empirical distributions from prior real-world measurements of enterprise application traffic [21].

This synthetic traffic generation method closely follows the patterns described in standardized workload simulation studies and ensures

- Randomized temporal distribution, avoiding periodicity;
- Statistical variation in intensity and payload, simulating bursty and heterogeneous demand;
- Protocol consistency, using standardized HTTP/1.1 over TCP with client-initiated transactions.

This model does not rely on Poisson arrivals per se but instead uses a configurable pseudo-random process, implemented via Python 3.12.7’s random module, leveraging the industry-standard Mersenne Twister algorithm. This deterministic PRNG offers robust statistical properties and a prolonged period, critical for reproducibility and high-quality sequences. Traffic generation utilizes both uniform and empirical distributions (via random.uniform and random.choices, respectively), which offers greater control over traffic burstiness and approximates variable-rate workloads seen in real deployments. Our idea is to ensure reproducibility, flexibility, and a realistic approximation of enterprise-grade traffic, providing a robust foundation for evaluating the performance and adaptability of our dynamic load-balancing framework. The traffic sources and their roles are summarized in Table 4.

### 4.4. Testing Scenarios

To test the robustness and adaptability of our proposed AADLB algorithm, we defined various testing scenarios that simulated typical enterprise environments. We benchmarked it with WRR, WFQ, PQ, and DRR as traditional load-balancing schemes commonly used in SD-WAN and enterprise deployments. The algorithms were tested using the previously mentioned traffic generator and topology. All instances were exposed to identical traffic patterns over 110 min. The behavior of each algorithm was independently measured and compared using identical performance metrics, enabling a fair and reproducible evaluation across all load-balancing schemes. The metrics, including CPU and RAM utilization, response time, connection error rate, and instance weights (when applicable), were continuously recorded.

- Test Scenario 1—Backend Instance Failure: To replicate a real-world failure condition, one backend instance (Instance 3) was manually taken offline between minutes 30 and 40. During this period, we measured CPU and RAM utilization, connection durations, and error rates. The goal was to evaluate system response and check each algorithm’s ability to reroute traffic and restore service after reintegrating the failed instance.
- Test Scenario 2—Load Distribution and Latency Due to Service Placement: To analyze sensitivity to inter-WAN delays, the central MySQL database was hosted in WAN-1. Traffic was then analyzed to compare instances located within the same WAN (low latency) versus those in different WANs (higher latency). App instances on different WAN networks should have a longer average connection time and a higher error rate compared to those on the same network as the database. This will impact how the weighting algorithm allocates the traffic. This way, we can assess our algorithm’s sensitivity to network topology and inter-WAN latencies.

This evaluation framework compares the strengths and weaknesses of traditional methods with the adaptive features of our new AADLB algorithm.

## 5. Results and Analysis

Over a 110 min test, we evaluated our proposed AADLB algorithm, WRR, WFQ, PQ, and DRR algorithms by measuring CPU and RAM utilization, weight assignments, and connection response times across four service instances at 1 min intervals. At minutes 30 and 40, Instance 3 was deliberately taken offline, resulting in 0% utilization, and the system automatically redistributed traffic among the remaining instances. When Instance 3 was brought back online at minute 40, its weight (and thus utilization) spiked quickly before stabilizing. In this research, AADLB was benchmarked against both static and semi-static schemes. WRR uses fixed, manually assigned weights to distribute traffic cyclically, providing basic fairness but no runtime adaptability. PQ assigns strict priorities to queues based on deployment-time policies and does not adjust dynamically, often leading to lower-priority starvation under heavy load. WFQ approximates the ideal Generalized Processor Sharing (GPS) model by assigning each flow a weight and scheduling packets based on their virtual finish time. This mechanism ensures weight fairness, but the weights are typically configured statically and do not adapt to fluctuating resource availability or performance changes at runtime. DRR enhances round robin fairness by introducing a deficit counter, which tracks leftover credits for each flow. In each round, a flow is allowed to transmit packets if the sum of their sizes does not exceed its current deficit counter value. The counter is incremented each round by a fixed quantum, assigned statically at configuration time. This approach supports flows with variable-size packets without requiring per-packet weight recalculation, but it lacks dynamic adjustment of quantum values to reflect real-time system conditions. Although other algorithms like Round Robin (RR) were initially considered, RR was excluded, as it does not support weighted scheduling at all. WRR, WFQ, and DRR were chosen as more relevant baselines, since they incorporate weight or fairness considerations. Overall, our algorithm’s core contribution lies in dynamic adaptive weighting, continuously recalculating weights based on live performance and resource usage metrics to achieve better responsiveness, fairness, and system utilization compared to static or semi-static methods.

**Figure 4:**
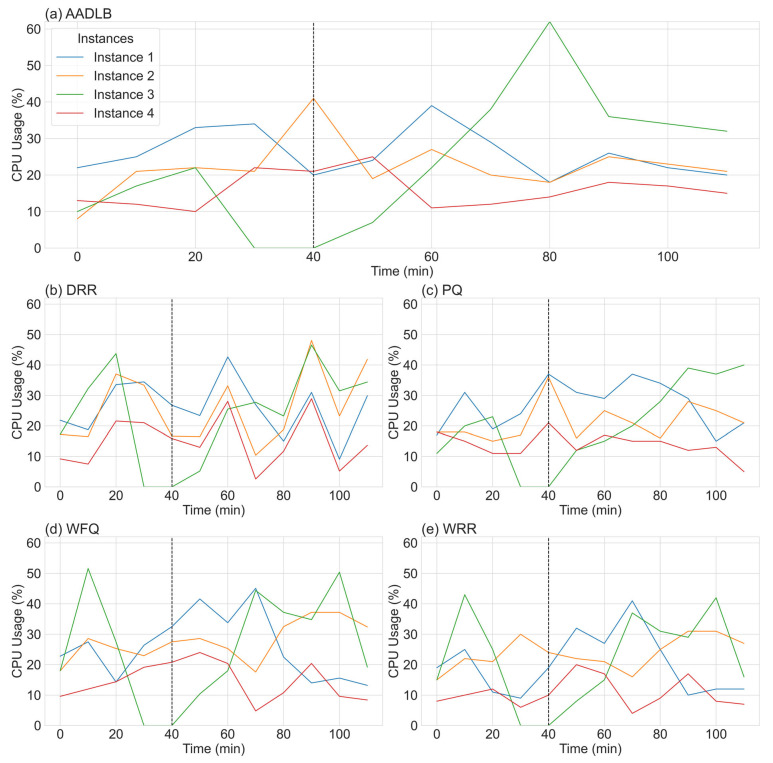
CPU utilization graph (%).

**Figure 5:**
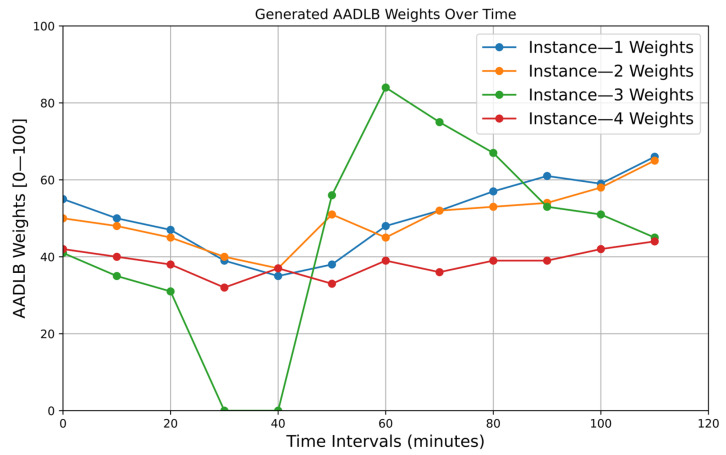
AADLB weights generated over time.

Before turning to the full-scale statistical comparison, we begin with two illustrative time–series plots (Figure 4 and Figure 5) captured from a single representative run of the experiment. These side-by-side graphs serve a dual purpose. First, they reveal in real time how each of the five schedulers—our adaptive AADLB together with the reference DRR, WFQ, WRR, and PQ—responds to an abrupt node failure and its subsequent recovery. Second, they expose the internal dynamics by which the adaptive scheme recalculates weights and redistributes load during the disturbance. By following these concrete traces step by step, we can see how AADLB’s network-aware adjustments smooth resource utilization, whereas the fixed quantum of DRR, the fluid shares of WFQ, the round robin cycle of WRR, and the rigid priorities of PQ can leave spare capacity idle or trigger volatile spikes.

The five subplots in Figure 4 track the CPU utilization of four service instances under five different schedulers—our adaptive AADLB, alongside the reference algorithms DRR, WFQ, WRR, and PQ—throughout a 110 min experiment. A critical failure was intentionally triggered at t = 30 min, when Instance 3 was taken offline; the outage window lasted until t = 40 min. The end of this failure period is indicated in each subplot by a vertical dashed line. The schedulers’ reactions revealed significant performance differences. AADLB responded immediately and effectively; as the AADLB Instance 3 trace dropped to 0%, its controller rerouted traffic in real time, causing CPU load on AADLB Instance 2 and AADLB Instance 4 to climb by 19 and 11 percentage points, respectively, by minute 40. In stark contrast, the responses of the other schedulers were significantly less effective. While DRR, WFQ, WRR, and PQ did attempt to reallocate traffic, their response was sluggish and inefficient, failing to achieve the immediate and targeted load transfer demonstrated by AADLB. When AADLB Instance 3 came back online at t = 50 min, AADLB again demonstrated its sophistication by assigning it a temporary weight boost to compensate for its downtime. This resulted in a controlled surge in AADLB Instance 3 utilization, which peaked at around 60% at t = 80 before quickly stabilizing with the other nodes. The recovery of the traditional schedulers was far less efficient: WRR’s was slow and linear, while PQ, DRR, and WFQ exhibited volatile oscillations and overshoots, struggling to smooth demand across the cluster. Overall, the data reinforce AADLB’s key advantages: its fast failure detection, effective load transfer, and rapid re-stabilization decisively outperform the traditional schedulers in this scenario.

Figure 5 visualizes the internal weighting decisions that drive AADLB’s scheduling logic. Until minute 30, all four instances hold moderate, roughly balanced scores (40–55 points), reflecting similar performance and latency. After Instance 3 is taken offline, its weight is immediately clamped to zero, while the remaining instances’ weights edge downward despite carrying extra traffic; the controller deliberately lowers their scores to acknowledge the stress they are already under and to avoid oversaturation. When the recovered instance re-registers at minute 40, the algorithm reacts aggressively: AADLB Instance 3 weight leaps from 31 to 56 and then to 84 by minute 60, directing a burst of sessions its way, so that backlog and cache-warm-up effects clear quickly.

As its utilization converges with the rest of the fleet, the weight decays toward the 45–50 band, bringing the system back to equilibrium. Instances 1 and 2, which enjoy lower round-trip times to the database, retain slightly higher steady-state weights than Instance 4, whose persistent mid-30 s score reflects marginally poorer latency. WRR is excluded from the figure because its weights are static configuration parameters that never change. Similarly, DRR and WFQ weights are also static and do not change dynamically based on real-time metrics. PQ has no numeric weights at all, only fixed priority classes. Therefore, the introduction of any of these algorithms to the plot would result in straight, unchanging lines that do not contribute meaningful dynamics to a weight-over-time graph.

**Figure 6:**
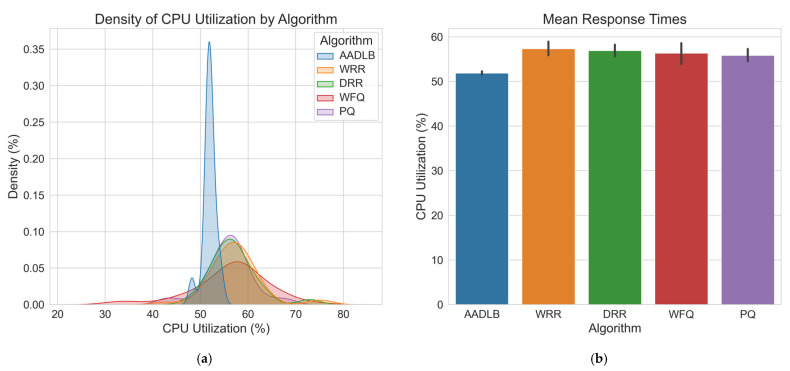
CPU utilization graph. (**a**) Illustrates CPU utilization, with the *X*-axis spanning roughly between 20% and 85% (peak density occurring between 35% and 75%) and the *Y*-axis showing density, the relative probability of observing each utilization level; (**b**) Displays mean CPU utilization with standard deviation error bars (*X*-axis: Algorithm, *Y*-axis: CPU utilization [%]), providing a comparative view of average processor load for each algorithm.

**Figure 7:**
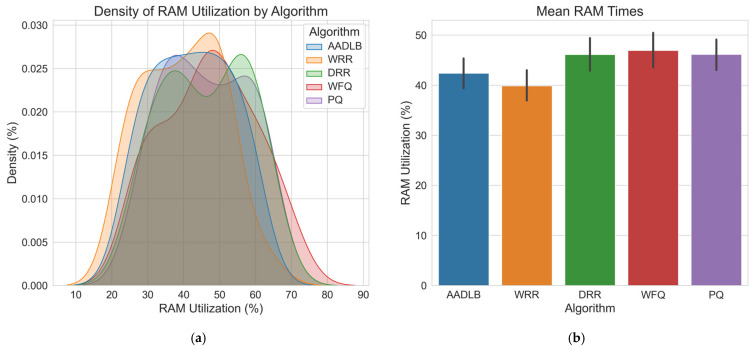
RAM utilization graph. (**a**) Illustrates RAM utilization, with the *X*-axis showing RAM percentage (roughly between 10% and 80%, most values between 25% and 70%) and the *Y*-axis representing the density, that is, the relative probability of observing each utilization level; (**b**) Presents mean RAM utilization with standard deviation error bars (*X*-axis: Algorithm, *Y*-axis: RAM utilization [%]), enabling direct assessment of average memory load across algorithms.

**Figure 8:**
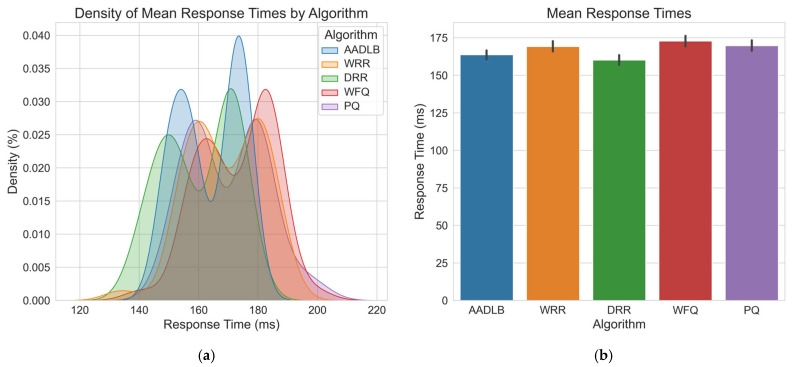
Connection response time graph. (**a**) Presents the response time distribution, with the *X*-axis listing response times from roughly 120 ms to 220 ms and the *Y*-axis showing density, the relative probability of observing each response time value rather than a raw count of occurrences; (**b**) Displays the mean response times with standard deviation error bars (*X*-axis: Algorithm, *Y*-axis: Response time [milliseconds]), enabling direct comparison of average latency across AADLB, WRR, DRR, WFQ, and PQ.

Having visually demonstrated the adaptive capabilities and internal mechanics of our proposed AADLB algorithm through the time–series plots in Figure 4 and Figure 5, we now proceed to a comprehensive statistical evaluation. The preceding analysis established that AADLB effectively responds to dynamic system changes, reallocating load smoothly and efficiently, in contrast to the limitations of static or semi-static baseline approaches. To ensure robustness and minimize anomalous variations, the data for this comparison were generated by averaging the results from three identical test runs. The subsequent analysis presents a detailed quantitative comparison of the algorithms, utilizing histograms for CPU, RAM, and response time distributions (Figure 6, Figure 7 and Figure 8), along with comprehensive statistical tables to rigorously assess their efficiency and stability.

As shown in Figure 6, the highest number of measurements (represented by the peak of the histogram) falls within the range of 50–60% CPU usage. AADLB has by far the most occurrences in the 50–55% range, which means it uses the CPU efficiently and stably. The WRR and DRR curves have modest tails that reach into the 65–70% region, but their highest density, such as AADLB’s, remains in the 55–60% band. These occasional excursions suggest sporadic, not sustained, periods of elevated CPU load. WFQ and PQ display broader, flatter distributions: their peak density still lies in the 55–60% band; yet, both curves extend with low-probability tails into the 65–70% region. This shape suggests occasional but not sustained episodes of higher CPU load, indicating more variable utilization compared with AADLB. The bar chart on the right side of Figure 6 summarizes the mean CPU load for each algorithm. It shows that AADLB averages around 52%, whereas the other four schedulers cluster between 55% and 58%. This numerical view corroborates the density curves: AADLB sustains the lowest and most stable utilization, while the alternative algorithms operate at consistently higher averages.

Figure 7 shows that all five RAM utilization curves overlap most densely between ≈30% and 60%, with only light tails extending beyond this band; therefore, no scheduler dominates the entire range. DRR, WFQ, and PQ produce such tails above 50%; yet, their peak density still sits in the 45–50% zone. WRR and DRR cover the broadest span, hinting at occasional workload swings, whereas PQ clusters in the mid-forties, and WFQ maintains a lower, flatter profile. The bar chart on the right confirms these findings: WRR records the lowest average RAM utilization at around 40%; yet, AADLB, while slightly higher at 43%, exhibits the narrowest spread across the KDE curve, indicating far more stable memory usage with smaller fluctuations. Taken together with the CPU results, this suggests that AADLB provides the most consistent resource footprint, while WRR and DRR are less predictable, and PQ and WFQ occupy a middle ground.

Figure 8 shows that most response times are between 150 ms and 180 ms. This is the most common range for all algorithms. AADLB has a high frequency in the range of 155–170 ms, which means it gives the most stable response times. Its distribution is narrow and high, which shows consistency. WRR exhibits a wider spread in response times, signaling higher variability, whereas DRR’s distribution is comparatively narrow and centered around 150–160 ms, indicating more consistent performance. The WFQ and PQ algorithms are present in the entire range, including higher response values (180–200 ms), indicating that they can be slower in some cases. The accompanying bar chart confirms that DRR delivers the lowest average response time at about 155 ms; yet, AADLB, only slightly higher at roughly 160 ms, exhibits the narrowest spread, indicating the most consistent latency profile. All of this indicates that AADLB shows the most stable responses, which is very important in real-time or sensitive systems. Other algorithms (WRR, WFQ, PQ) show wider ranges, which may indicate more unstable performance. We believe that if the goal is speed and reliability of response, AADLB currently provides some improvements.

Table 5 shows that the AADLB algorithm has the second fastest average response time of 163.69 ms, right after DRR. It also has the smallest standard deviation (10.30 ms) and shows the most stable and predictable performance. The minimum response value is 148.8 ms in the upper half, but not extremely low. The maximum response is 176.6 ms, and it is the lowest among all algorithms, except DRR. On the other hand, DRR has a slightly faster average response but with higher variability and lower minimum (it is less consistent). WRR, PQ, and WFQ are slower and less reliable, with higher average and larger oscillations. Thus, AADLB offers a statistically significant latency advantage over WRR, WFQ, and PQ and a stability advantage over DRR. Although DRR has a slightly lower average, AADLB is more stable and consistent, which is desirable in systems where predictability is more important than extreme performance. Future updates will include hardware-independent indicators like requests-per-second per core or memory pressure signals, ensuring usability in diverse or cloud-based environments.

It is important to emphasize that host-level metrics, such as CPU and RAM utilization, are intentionally computer-dependent. The algorithm’s core innovation precisely leverages these host-specific metrics to optimize resource allocation. Nevertheless, network-level metrics like average response time and data throughput, while influenced by infrastructure, provide complementary insights crucial for comprehensive system evaluation.

The experimental results demonstrate clear, practical improvements in system performance when using the AADLB framework compared with traditional load-balancing methods. Average CPU utilization and end-to-end latency were computed from the 10 min average results captured for each algorithm, and variability was quantified with the sample standard deviation (σ). On this basis, the AADLB algorithm improved average CPU utilization by 8.40%, markedly lowered CPU utilization variability (stability) by 76.66%, reduced RAM utilization variability (stability) by 6.97%, slightly decreased average latency by 2.58%, and tightened latency spread by 16.74%. Pairwise differences in latency were evaluated with Welch’s unequal-variance *t*-test. These tests confirmed that AADLB provided statistically significant latency improvements when compared directly with WRR (*p* = 0.019), WFQ (*p* = 1.4 × 10^−4^), and PQ (*p* = 0.012). Although the latency difference between AADLB and DRR was not statistically significant (*p* = 0.13), AADLB exhibited superior variability control, outperforming DRR and all other methods in CPU utilization variation (σ = 1.36%) and response time variation (σ = 10.30 ms). RAM utilization differences showed marginal statistical significance (best *p*-value = 0.065 compared with WFQ), suggesting variability reductions that, while promising, warrant further experimental validation. Overall, considering that even modest enhancements in latency, resource utilization, and variability can substantially impact service quality and operational costs, these statistically verified improvements underscore the practical and operational relevance of our proposed algorithm. Future work involving larger datasets and extended experimental scenarios is recommended to further validate and possibly enhance these findings.

## 6. Conclusions

This research presents an adaptive, Application-Aware Dynamic Load-Balancing (AADLB) framework for open-source SD-WAN environments. Unlike traditional mechanisms (e.g., round robin, least connections, or static weight-based methods), which overlook application-specific requirements and runtime network dynamics, the proposed approach integrates host-level and connection-level telemetry into routing decisions. This methodological improvement enables fine-grained, context-aware traffic distribution and enhances performance and responsiveness under fluctuating conditions. The core contribution lies in a heuristic-based weight assignment algorithm that dynamically adjusts traffic flows based on CPU and RAM utilization, connection trends, error rates, and response durations. This approach helps reduce congestion, ensure service continuity, and keep stable performance even during node failures or peak loads.

Experimental evaluation in a realistic SD-WAN testbed confirms the effectiveness of the proposed framework. Compared to WRR, PQ, WFQ, and DRR algorithms, AADLB achieved an 8.40% improvement in average CPU utilization, a 76.66% increase in CPU stability, a 6.97% gain in RAM stability, a 2.58% reduction in average response latency, and a 16.74% improvement in latency consistency. These performance gains validate the operational value of the framework for latency-sensitive services, such as VoIP, video conferencing, and transactional APIs, while also demonstrating cost efficiency and scalability for enterprise deployments.

Beyond technical performance, the framework addresses key limitations of traditional SD-WAN solutions, including static traffic allocation, lack of application awareness, and vendor lock-in. By eliminating reliance on proprietary hardware or rigid configuration policies, it offers a flexible, transparent, and extensible alternative tailored to open-source ecosystems.

Future work will focus on augmenting the framework with predictive analytics and reinforcement learning to enable proactive traffic steering and self-tuning capabilities. Planned validation in larger-scale multi-cloud topologies and the integration of advanced fault tolerance and security mechanisms will further demonstrate its applicability. Overall, this research lays the foundation for a new generation of intelligent, responsive, and open SD-WAN systems. The source code is available for everyone. It can help the community create new additions, test performance, and work together on new ideas.

## Figures and Tables

**Table 1:** Proprietary vs. open-source SD-WAN: Advantages and constraints.

SD-WAN Platform	Advantages	Constraints
Cisco SD-WAN	Integration with Cisco devices	High cost of licenses and support
	Advanced functionalities (traffic optimization, security)	Complicated configuration
	Centralized management (vManage)	Dependence on the Cisco ecosystem
VeloCloud (VMware SD-WAN)	Simplified management through the VMware platform	Scalability in large networks
	Intelligent traffic routing Scalability in large networks Integration with VMware solutions	Integration with VMware solutions
Fortinet SD-WAN	Integration with FortiGate firewalls	Scalability in large networks
	Advanced security functionalities (IPS, VPN, antivirus)	Integration with VMware solutions
	Traffic optimization (QoS, load balancing)	Scalability in large networks
	Centralized management (FortiManager) Scalability in large networks Integration with VMware solutions	Integration with VMware solutions
OpenWrt	Open solution Flexibility and adaptability Large community and support Easy customization of functionality	Complicated setup Limited advanced functionality Lack of official support Need for technical expertise
VyOS	Free solution Flexibility and adaptability Compatibility with different devices and networks	Limited functionality compared to proprietary solutions Lack of official support Difficult to implement and configure
pfSense	Free and open-source	Technical knowledge required for implementation
	Strong security and VPN options Compatibility with various network devices Large community	Limited scalability compared to proprietary solutions

**Table 2:** Performance metrics for dynamic load balancing.

Metric	Description and Functional Role
CPU Utilization	Measures the processing load on each backend instance. High CPU usage decreases the instance’s likelihood of receiving new traffic.
RAM Utilization	Indicates memory pressure. Instances with elevated memory usage may respond slower and are deprioritized in load distribution.
Trend Coefficient	Reflects the temporal change in CPU and RAM utilization over a defined interval (e.g., 1 min). A positive trend suggests increasing load, while a negative trend implies recovery. This metric enables proactive traffic redirection.
Average Connection Duration	Represents how long requests stay active. Longer durations often indicate performance degradation or saturation and reduce the instance’s routing weight.
Bytes Sent and Received	Quantifies bandwidth usage. Sustained high throughput may indicate either high efficiency or network saturation and is interpreted in conjunction with other metrics.
Connection Error Ratio	Tracks the percentage of failed connection attempts. A rising error ratio signals instability or overload and results in penalizing the instance within the routing decision process.

**Table 3:** VM topology and resource allocation in the SD-WAN test environment.

Component	Count	Resources (vCPU/RAM)	Purpose
Application Instances	4	2 vCPU/2 GB	Backend APIs
Database Server	1	2 vCPU/2 GB	Central data storage (WAN-1)
Load Balancer	1	4 vCPU/8 GB	Fabio
Controller	1	6 vCPU/16 GB	SD-WAN management
Monitoring and Logging	2	4 vCPU/8 GB	Zeek, Logstash, Filebeat, Elasticsearch
Traffic Generators	3	2 vCPU/4 GB	Simulated user request generation

**Table 4:** Traffic types by source.

Node	Network Domain	Purpose	Traffic Type
LB	Central Hub	Load Balancer	Receives and forwards traffic
Controller	Central Hub	Controller, Data Processor	Central data storage
Log Server	Central Hub	Log Server	Monitoring, Logging
APP-1	WAN-1	App Instance 1	REST API, Database Access
APP-2	WAN-1	App Instance 2	REST API, Database Access
DB	WAN-1	MySQL Database	Bulk SQL Transactions
APP-3	WAN-2	App Instance 3	REST API, Database Access
APP-4	WAN-2	App Instance 4	REST API, Database Access
TG1	External	Traffic Generator 1	REST Requests
TG2	External	Traffic Generator 2	WebRTC Requests (UDP)
TG3	External	Traffic Generator 3	SQL (Database Queries)

**Table 5:** Statistical analysis of the response time by algorithm.

Algorithm	Avg Response Time (ms)	Standard Deviation	Min Response Time (ms)	Max Response Time (ms)
DRR	160.21	12.05	138.9	178.5
AADLB	163.69	10.30	148.8	176.6
WRR	169.23	12.31	134.0	188.0
PQ	160.77	12.87	148.1	200.6
WFQ	172.90	12.26	140.6	199.2

## Data Availability

The datasets used and analyzed in the current study are available from the corresponding author upon reasonable request.
